# Interaction between blood pressure and genetic risk score for bladder cancer, and risk of urothelial carcinoma in men

**DOI:** 10.1038/s41598-022-23225-6

**Published:** 2022-10-31

**Authors:** Stanley Teleka, Marju Orho-Melander, Fredrik Liedberg, Olle Melander, Karin Jirström, Tanja Stocks

**Affiliations:** 1 Department of Surgical Sciences, Uppsala University, Epihubben, Dag Hammarskjölds Väg 14 B, 75185 Uppsala, Sweden; 2 Department of Clinical Sciences in Lund, Lund University, Lund, Sweden; 3 Department of Clinical Sciences in Malmö, Lund University, Lund, Sweden; 4 Department of Urology, Skåne University Hospital Malmö, Malmö, Sweden; 5 Department of Translational Medicine, Lund University, Malmö, Sweden

**Keywords:** Health care, Risk factors

## Abstract

There is substantial genetic predisposition to bladder cancer (BC). Recently, blood pressure (BP) was positively associated with BC risk in men, but the potential interaction with genetic susceptibility for BC is unknown. We investigated a weighted genetic risk score (wGRS) of 18 BC genetic variants, BP, and their interaction, in relation to incident urothelial cancer (UC, n = 385) risk in 10,576 men. We used Cox regression, the likelihood ratio test, and the relative excess risk for interaction to calculate hazard ratios (HR) of UC, multiplicative interaction and additive interaction respectively. There was evidence of a positive additive interaction between SBP and the wGRS in relation to aggressive (P = 0.02) but not non-aggressive (P = 0.60) UC. The HR of aggressive UC was for SBP ≥ 140 mmHg and the upper 50% of the wGRS combined 1.72 (95% CI 1.03–2.87) compared to the counterpart group. Additionally, the 20-year risk of aggressive UC in 60 year-old men was 0.78% in the low SBP/low wGRS group and 1.33% in the high SBP/high wGRS group. Our findings support a potential additive interaction between the wGRS and SBP on aggressive UC among men. If replicated, the findings on interaction may provide biological and public health insight to prevent aggressive UC.

## Introduction

Urothelial carcinoma (UC) is a cancer that originates from the mucosal surfaces (termed “urothelium”) of renal calyces, pelvis, ureters, bladder and urethra. Urothelial bladder cancer (BC) has by far the highest frequency of occurrence, comprising between 90 and 95% of all UC^1^. BC is a heterogeneous disease with known genetic and environmental risk factors^2,3^. With regards to genetic predisposition, 31% of BC cases are estimated to be attributed to genetic variation, and previous studies have reported a twofold increased risk among first degree relatives with BC^4–6^. While rare germline mutations with strong effects on disease risk, such as the DNA-mismatch repair protein 2 (*MSH2*) mutation in Lynch syndrome have been found^7^, the genetic mechanisms behind a majority of BC is assumed to be polygenic, whereby individual genetic variants each have a small effect on disease risk^8^. Single nucleotide polymorphism (SNP) is the most common type of genetic variation in humans, and at least 28 SNPs related to BC have been discovered, most through genome-wide association studies (GWAS)^7,9^. In a polygenic disease, where a single variant may not be informative, a genetic risk score (GRS) can be generated to sufficiently identify those at high risk^10^.

The association between blood pressure (BP) and cancer is an area of investigation that has received increased attention in recent times. The most consistent evidence linking BP to a site–specific cancer is for renal cell carcinoma^11^. With respect to BC, evidence from the largest prospective studies, including our own studies, report a positive association only among men, and a stronger association with muscle invasive BC (MIBC)^12–15^.

Gene-environment interaction may provide insight into biological mechanisms of a disease, and can be assessed on an additive and multiplicative scale^16^. BC, being a disease with complex etiology, is an ideal setting to investigate the complex interplay between genetic and environmental risk factors^3^. The most established gene-environment interaction in relation to BC includes smoking and N-acetyltransferase 2 (*NAT2*), and smoking and glutathione S-transferase-mu 1 (*GSTM1*)^6,8,17^. Other environmental risk factors investigated in such interactions include occupational carcinogens and caffeine^2,18–20^. The potential interaction between BP and genetic susceptibility in relation to BC or UC has not been investigated. Herein, we investigated a bladder cancer GRS, BP, and their interaction, in relation to UC risk, overall and separately for aggressive and non-aggressive tumors, in men.

## Methods

### Study population

This study included participants from the Malmö Diet and Cancer Study (MDCS), a population-based prospective cohort study from Malmö, a city in southern Sweden. The cohort included 30,447 men and women aged between 45 and 73 years, who underwent a baseline health examination between 1991 and 1996. A full description of the cohort is published elsewhere^21^. The cohort made up 60% of the Swedish population (46% of cases) in our previous pooled study of BP and BC risk^13^.

### Ethical approval

This study was performed in accordance with the Declaration of Helsinki (2008). All participants provided a written informed consent at baseline physical examination to have their data used for research. Furthermore, all methods were carried out in accordance with the guidelines and regulations of the Lund University Research Ethics Committee who also approved the study of the MDCS (STYR 2019/2046).

### Exposure assessment

A standard mercury sphygmomanometer placed on the right arm was used to obtain the BP levels. BP was taken twice in a supine position with a rest of 5 min between the readings. The average value between the two readings was then reported as the actual BP level. BP was measured and recorded for each individual at the time of baseline examination. To obtain BMI, height and weight were taken with no shoes and only with light indoor clothing. Information on smoking habits, physical activity during leisure time, and highest level of attained education was obtained from a questionnaire asked at the baseline health examination^21^.

### Selection of SNPs and genotyping

Genetic variants associated with BC were identified from published genome-wide association studies, which extend from 2008 to 2017^7,22^ (Table 1).

**Table 1:** List of bladder cancer single nucleotide polymorphisms (SNPs) used to create the weighted genetic risk score.

SNP	Gene	Effect allele	Other allele	EAF (%)	Odds ratio	ln (Odds ratio)
rs1014971	*CBX6/APOBEC3A*	T	C	65.6	1.18	0.165514
rs10775480	*SLC14A2*	T	C	45.5	1.13	0.122218
rs10936599	*ACTRT3/MYNN/TERC/LRRC34*	C	T	74.2	1.15	0.139762
rs11892031	*UGT1A8/UGT1A10*	A	C	91.9	1.17	0.157004
rs1495741	*NAT2*	A	G	72.7	1.13	0.122218
rs17674580	*SLC14A1*	T	C	36.9	1.17	0.157004
rs2294008	*JRK/PSCA*	T	C	42.4	1.13	0.122218
rs2736098^a^	*TERT*	A	G	31.3	1.16	0.14842
rs401681	*TERT/CLPTM1L*	C	T	57.8	1.12	0.113329
rs4907479	*NR*	A	G	27.8	1.13	0.122218
rs6104690	*C20orf187/ LOC339593*	A	G	42.4	1.07	0.067659
rs62185668	*JAG1*	A	C	25.8	1.19	0.173953
rs710521	*TP63*	A	G	70.2	1.18	0.165514
rs7238033	*SLC14A1*	C	T	45.5	1.2	0.182322
rs798766	*TMEM129/TACC3/FGFR3*	T	C	79.3	1.2	0.182322
rs8102137	*CCNE1*	C	T	28.8	1.13	0.122218
rs907611	*LSP*	A	G	33.3	1.15	0.139762
rs9642880	*MYC/BC042052/CASC11*	T	G	40.9	1.21	0.19062

SNP, single nucleotide polymorphism; EAF, effect allele frequency; ln, natural log.

SNP information (except rs2736098 [*TERT*]) was obtained externally from genome-wide association studies of BC reviewed in: de Maturana, E. L. *et al.* Bladder cancer genetic susceptibility. A systematic review. *Bladder Cancer*. **4**, 215–226. https://doi.org/10.3233/blc-170159 (2018)^7^.

^a^SNP information on rs2736098 (*TERT*) was obtained from the GWAS catalog: Institute, N. H. G. R. (2008). www.ebi.ac.uk/gwas/home.

Eighteen SNPs included in this study had been discovered and validated in a population of European ancestry, and SNPs discovered through other study designs/methods, and from populations of other ancestries were not included. Genotyping for the study participants was performed using the Illumina GSA v1 genotyping array. An internal quality control check excluded samples with a low call rate (< 90%), SNPs that were out of Hardy–Weinberg equilibrium, and those that exhibited discordance between reported and genetically inferred sex^23^. The Haplotype Reference Consortium, a large reference panel of human haplotypes was used to perform the genotype imputation^24^.

To generate a weighted GRS (wGRS) of the 18 SNPs, the genotype dosage for each SNP (coded as 0, 1 and 2 for each risk increasing allele) was multiplied by its respective weight (beta-coefficient from the association of each SNP with BC) obtained from GWAS of BC, followed by summation across all the variants according to the following equation*** (wGRS for each individual = [β_1_ × SNP_1_ + β_2_ × SNP_2_ + …β_x_ × SNP_x_]/number of SNPs). For GWAS of BC that expressed the association between SNP and BC as odds ratios, the natural log (ln) was used to convert the odds ratio to beta-coefficient.

### Follow-up and end-point assessment

Any diagnosis of cancer, cause of death and emigration status were identified through linkage of each study participants’ unique civil registration number with the National Cancer Register, Cause of Death Register, and Population Register, respectively. Follow-up of these linkages ended on 31 December 2018. UC was defined according to the seventh edition of the International Classification of Diseases (ICD-7) codes 180.1 and 181 (ICD-10, C65-68 [0–9], including carcinoma in situ D09 [0–1]). All specimens taken from the UC cases underwent histopathological re-evaluation. The stage of the primary tumor (pT) was based on the TNM 2017. We classified/stratified tumor aggressiveness based on whether the tumor invaded the muscularis propria layer and UC-specific mortality. Non-aggressive tumors included non-muscle invasive (Ta, Tis, and T1) tumors that had not led to mortality due to UC, and aggressive tumors included muscle-invasive (T2-T4) tumors and any UC recorded as the underlying cause of death. We initially considered to define tumor aggressiveness by including Tis and T1 tumours in the “aggressive” group; however, we opted for muscle-invasiveness as the base for classification because these groups have shown a greater difference in association with BP and wGRS (hazard ratios) and with UC-specific mortality plotted with Kaplan–Meier curves^25^.

### Selection criteria

**Figure 1:**
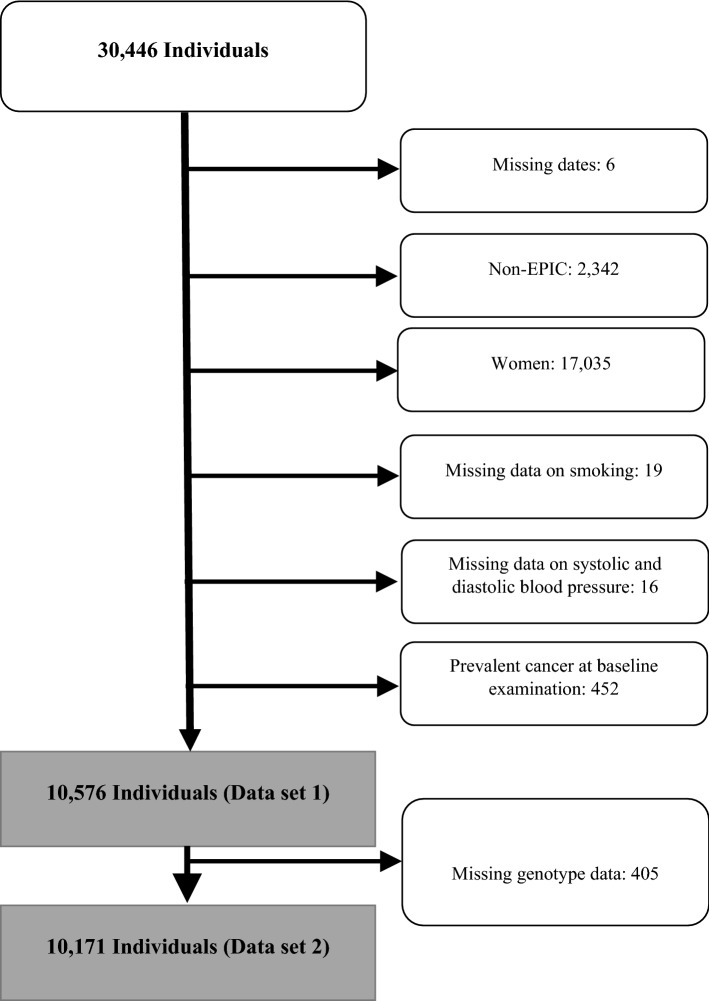
Flow chart showing the selection of the study participants. Data set 1 was used for associations between the blood pressure indices and urothelial carcinoma (UC) outcomes. Data set 2 was used for association between the weighted genetic risk score for bladder cancer and UC outcomes and the interaction analysis.

From a study population of 30,446 participants, only the Malmö-EPIC sub-population was included, from which 10,576 men were included in the final analysis (Fig. 1). Female sex was the main cause of exclusion (n = 17,035). The reasons for excluding women in the analysis were a sex-interaction with SBP (p-value = 0.04 for aggressive UC) and no association between BP and BC risk in women in the largest prospective studies^12,15^, and low statistical power (177 incident UC cases) for a separate analysis of women. After follow-up and histopathological re-evaluation of tumors, we identified 385 incident UCs (365 BCs), out of which 129 were categorized as aggressive and 246 as non-aggressive (10 UCs had missing tumor data).

### Statistical analysis

We calculated hazard ratios (HRs) and their 95% confidence intervals (95% CI) using Cox proportional hazards regression to investigate the association between BP (SBP and DBP separately), wGRS and UC risk (overall, and separately for aggressive and non-aggressive tumors). Age was used as the underlying time metric and participants were followed from the date of baseline health examination until the date of UC diagnosis, or until censoring due to migration or death, whichever one came first. The actual levels of SBP, DBP and wGRS were transformed to z-scores calculated as *z* = (*x − u*)/*σ*, where x is the actual level, *u* the mean, and *σ* the SD. Additionally, we investigated the associations based on categories for each exposure: SBP, < 140, 140–149, 150–159, ≥ 160 mmHg; DBP, < 90, 90–94, 95–99, ≥ 100 mmHg; and wGRS in quartiles. Models were adjusted for smoking in 5 categories (never-smokers, ex-smokers and tertiles of pack years in current smokers), BMI (quartiles), physical activity (tertiles), and level of education (5 categories). The p-value for trend across categories was investigated by incorporating the categories of SBP, DBP and wGRS as a continuous variable in the regression model and testing its coefficient using the Wald test. We tested for the Cox proportional hazards assumption using Schoenfield residuals, which showed no violation of the proportional hazards assumption.

**Figure 2:**
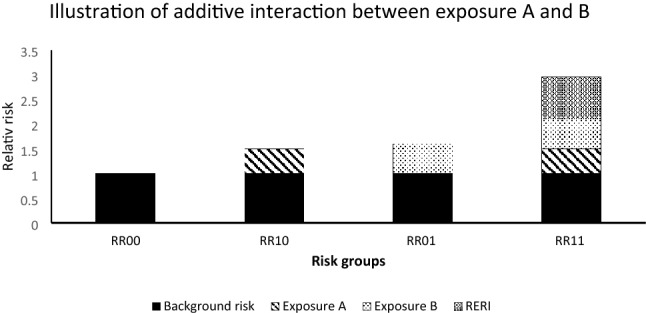
An illustration of additive interaction between Exposures A and B. RR_00_ represents the relative risk among those not exposed to A and B (also known as the background risk), RR_10_ represents the relative risk among those exposed to A only, RR01 represents the relative risk among those exposed to B only, RR11 represents the relative risk when exposed to both A and B, RR11 may additionally contain the relative excess risk of interaction (RERI), which is the excess risk that only occurs when exposure A interacts with exposure B additively.

To investigate the additive interaction between BP and wGRS in relation to UC, we tested whether the joint effect of BP and wGRS was larger than the sum of individual effects of BP and wGRS, as illustrated by Fig. 2. This was achieved by using the quantity “relative excess risk of interaction” (RERI) expressed as RR11 − RR10 − RR01 + 1, where: RR_00_ (or 1, reference group) represented individuals with normal BP (< 140/90) and lower 50% of the BC genetic risk; RR_10_ represented those with hypertension (≥ 140/90) and lower 50% of BC genetic risk; RR_01_ represented those with normal BP and upper 50% of BC genetic risk; and RR_11_ representing those with hypertension and upper 50% of BC genetic risk. When assessing additive interaction on a continuous scale, we used BP and wGRS expressed as z-score and limited the analysis to observations within ± 2 SD of the aforementioned exposures. The confidence intervals for the additive interaction were obtained using the delta method by Hosmer and Lemeshow^26^. To investigate the corresponding multiplicative interaction, we used the likelihood ratio test whereby the restricted model (without the product term) was nested in the model that additionally included the product term. Absolute risk for UC (between ages 60 and 80 years), with death as a competing event were calculated using a method described by Gail et al.^27^. All statistical analyses were performed in STATA 16 (StataCorp LLC, College Station, TX).

## Results

The participants were on average 59.0 (SD = 7.0) years old at baseline and were followed for 20.0 (SD = 6.9) years on average. During follow-up, 385 incident UCs, of which 248 non-aggressive and 127 aggressive, were recorded. Table 2 shows the characteristics of the participants separated by case status. Cases were more often current smokers compared with non-cases (41% for cases, 29% for non-cases).

**Table 2:** Characteristics of the 10,576 men in the Malmö Diet and Cancer Study.

Characteristic	Cases	Non-cases	Total
Population, n	385	10,191	10,576
Age at baseline, mean (SD)	60.4 (6.3)	59.0 (7.0)	59.0 (7.0)
**Categories, n (%)**			
< 50	22 (5.7)	1095 (10.8)	1117 (10.6)
50–54	68 (17.7)	2418 (23.7)	2486 (23.5)
55–59	78 (20.3)	2107 (20.7)	2185 (20.7)
≥ 60	217 (56.3)	4571(44.8)	4788 (45.2)
**Smoking status, n (%)**			
Never-smokers	50 (13.0)	2933 (28.8)	2983 (28.2)
Ex-smokers	179 (46.5)	4358 (42.8)	4537 (42.9)
Current smokers	156 (40.5)	2900 (28.4)	3056 (28.9)
**Pack years among current smokers, n (%)**			
< 10	22 (14.1)	494 (17.1)	516 (16.9)
10–19	24 (15.4)	343 (11.8)	367 (12.0)
≥ 20	110 (70.5)	2063 (71.1)	2173 (71.1)
**Blood pressure, mmHg, mean (SD)**			
Systolic blood pressure	146.0 (19.7)	143.8 (19.2)	143.8 (19.2)
Diastolic blood pressure	88.0 (9.6)	88.0 (9.9)	88.0 (9.8)
**Categories, systolic/diastolic, n (%)**			
< 140/90 mmHg	113 (29.4)	3552 (34.8)	3665 (34.6)
140/90–159/99 mmHg	161 (41.8)	3847 (37.8)	4008 (37.9)
≥ 160/100 mmHg	111 (28.8)	2792 (27.4)	2903 (27.5)
**Antihypertensive medication use**			
Yes	79 (20.5)	2079 (20.4)	2158 (20.4)
No	306 (79.5)	8112 (79.6)	8418 (79.6)
**Type of Antihypertensive medication among users, n**			
Diuretics	21	494	515
Beta blockers	45	1202	1247
ACE inhibitors	11	479	490
Calcium channel blockers	35	682	717
BMI, kg/m^2^, mean (SD)	26.7 (3.6)	26.2 (3.5)	26.3 (3.5)
**BMI, categories, n (%)**			
> 18.5	0 (0.0)	56 (0.6)	56 (0.5)
18.5–24.9	127 (33.0)	3794 (37.2)	3921 (37.1)
25–29.9	195 (50.6)	5030 (49.4)	5225 (49.5)
**≥ **30	63 (16.4)	1301 (12.8)	1364 (12.9)
Mean follow-up time, years (SD)	13.9 (6.9)	20.2 (6.8)	20.0 (6.9)
**Follow-up time, n (%)**			
< 5	52 (13.5)	448 (4.4)	500 (4.7)
5–9	68 (17.7)	735 (7.2)	803 (7.6)
10–14	80 (20.8)	1076 (10.6)	1156 (10.9)
≥ 15	185 (48.0)	7932 (77.8)	8117 (76.8)
**Education**			
Up to Elementary school	179 (46.6)	4665 (45.9)	4844 (45.9)
Up to lower secondary school education	76 (19.8)	1992 (19.6)	2068 (19.6)
Up to upper secondary school education	43 (11.2)	1215 (12.0)	1258 (11.9)
At least 1 year after secondary school	32 (8.3)	945 (9.2)	977 (9.3)
University degree	54 (14.1)	1351 (13.3)	1405 (13.3)

Majority of the participants used antihypertensive medication from different drug classes. BMI 10 missing; education 24 missing.

The associations between SBP, DBP, wGRS and UC outcomes are shown in Table 3. SBP was positively associated with aggressive UC risk (HR per SD, 1.28 [95% CI 1.08–1.52]), but not with UC overall and non-aggressive UC risk. There was a step-wise increased risk of overall and non-aggressive UC by increasing quartile level of the wGRS (p-trend < 0.01), and those in the fourth quartile of the wGRS had a significantly higher risk for UC overall (HR per SD, 1.66 [95% CI 1.25–2.20]) and non-aggressive UC (HR per SD, 2.06 [95% CI 1.44–2.95]) compared to those in the first quartile. The associations per SD wGRS were 1.27 (95% CI 1.15–1.40) for UC overall, 1.34 (95% CI, 1.18–1.52) for non-aggressive UC, and 1.19 (95% CI 1.00–1.42) for aggressive UC. There was no association between DBP and risk of UC outcomes.

**Table 3:** Hazard ratios and 95% confidence intervals for urothelial carcinoma outcomes by levels of systolic and diastolic blood pressure and a bladder cancer weighted genetic risk score.

Exposure	Analysis	Total UC	Non-aggressive UC	Aggressive UC
		Population, n (Cases, n)	Population, n (Cases, n)	Population, n (Cases, n)
		10,576 (385)	10,576 (248)	10,576 (127)
		HR (95% CI)	HR (95% CI)	HR (95% CI)
Systolic blood pressure	< 140 mmHg (reference)	1.00	1.00	1.00
	140–149 mmHg	0.98 (0.74–1.30)	0.89 (0.63–1.25)	1.33 (0.81–2.20)
	150–159 mmHg	1.12 (0.83–1.50)	0.90 (0.61–1.32)	1.80 (1.10–2.96)
	≥ 160 mmHg	1.16 (0.89–1.51)	1.05 (0.76–1.44)	1.50 (0.94–2.41)
	p-trend	0.23	0.85	0.06
	Per SD	1.09 (0.99–1.21)	1.00 (0.87–1.13)	1.28 (1.08–1.52)
Diastolic blood pressure	< 90 mmHg (reference)	1.00	1.00	1.00
	90–94 mmHg	1.11 (0.85–1.46)	1.06 (0.75–1.49)	1.19 (0.73–1.93)
	95–99 mmHg	1.05 (0.79–1.38)	1.15 (0.82–1.60)	0.85 (0.50–1.43)
	≥ 100 mmHg	0.99 (0.73–1.32)	0.76 (0.52–1.12)	1.51 (0.94–2.44)
	p-trend	0.89	0.27	0.16
	Per SD	1.00 (0.90–1.11)	0.93 (0.81–1.06)	1.17 (0.97–1.39)
Weighted genetic risk score	1st Quartile (reference)	1.00	1.00	1.00
	2nd Quartile	1.05 (0.77–1.44)	1.16 (0.78–1.74)	0.92 (0.54–1.56)
	3rd Quartile	1.26 (0.93–1.70)	1.33 (0.90–1.97)	1.24 (0.76–2.04)
	4th Quartile	1.66 (1.25–2.20)	2.06 (1.44–2.95)	1.24 (0.76–2.04)
	p-trend	< 0.01	< 0.01	0.26
	Per SD	1.27 (1.15–1.40)	1.34 (1.18–1.52)	1.19 (1.00–1.42)

*SD* standard deviation, *UC* urothelial carcinoma, *HR* hazard ratio, *CI* confidence interval.

Hazard ratios were calculated by Cox regression with age as the underlying time metric. Models were adjusted for categories of smoking, BMI, education, and physical activity.

10 individuals with incident UC cases had missing tumor data on muscle invasiveness.

405 (14 cases) individuals had missing genotype data (not included in analysis involving bladder cancer genetic risk score).

The p-value for trend across categories was investigated by incorporating the categories of SBP, DBP and wGRS as a continuous variable in the regression model and testing its coefficient using the Wald test.

**Figure 3:**
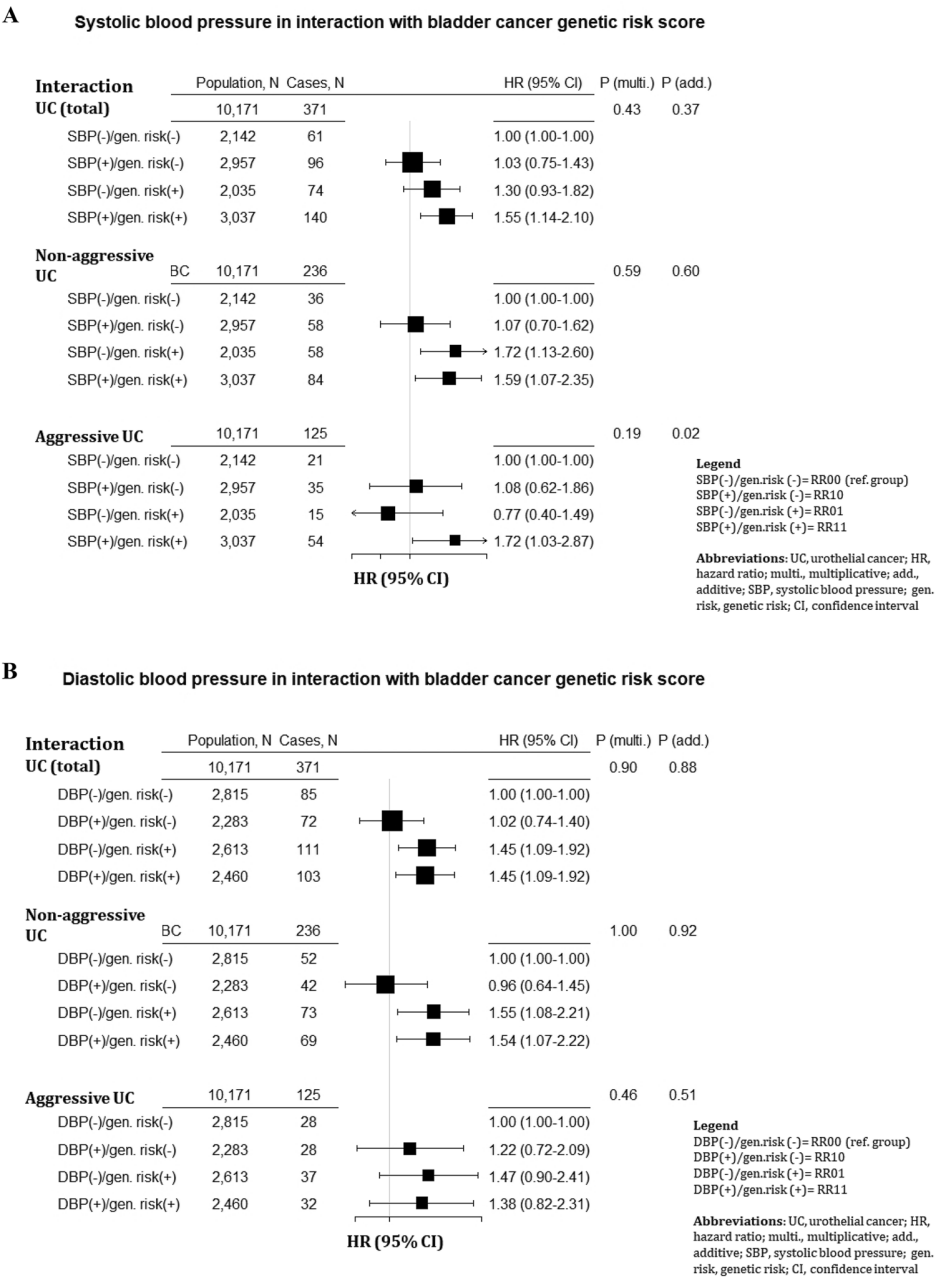
Hazard ratios (95% confidence interval) by groups of: (**A**) systolic blood pressure (SBP); and (**B**) diastolic blood pressure (DBP), and bladder cancer genetic score, including their multiplicative and additive interaction p-values, in relation to risk of urothelial cancer outcomes in the Malmö Diet and Cancer Study (MDCS; *N*_participants_ = 10,339; *N*_cases_ = 371). Hazard ratios were calculated by Cox regression with attained age as the underlying time scale, with adjustment for smoking, BMI, physical activity and level of education. The Relative excess risk for interaction (RERI) was calculated as RR_11_-RR_10_-RR_01_ + 1, where: RR_00_ (or 1, reference group) represented individuals with normal SBP/DBP (< 140/90) and lower 50% of the BC genetic risk; RR_10_ represented those with high SBP/DBP (≥ 140/90) and lower 50% of BC genetic risk; RR_01_ represented those with normal SBP and upper 50% of BC genetic risk; and RR_11_ representing those with high SBP and upper 50% of BC genetic risk. The confidence intervals for RERI were obtained using the delta method, the p-value for additive interaction (p-value [add.]) was obtained from the RERI model. Multiplicative interaction was calculated using the likelihood ratio test (LR test). P-value (multi.) is the p-value for the multiplicative interaction obtained from the likelihood ratio test.

Figure 3 shows HRs and additive and multiplicative interactions for combinations of SBP and DBP with the wGRS, with respect to UC outcomes. In relation to total and aggressive UC, high SBP (≥ 140 mmHg) combined with high wGRS composed the highest risk; HR per SD 1.55 (95% CI 1.14–2.10) and 1.72 (95% CI 1.03–2.87) respectively, compared to the low SBP-low wGRS group. There was a positive additive interaction between SBP and wGRS in relation to aggressive UC risk (RERI, 0.88 [95% CI 0.17; 1.58], p = 0.02), but not in relation to UC overall (p = 0.43) and non-aggressive UC risk (p = 0.60), and not for multiplicative interaction. To assess the robustness in our findings for aggressive UC, we repeated the analysis using 130 mmHg and 150 mmHg as the cut-off points for low and high SBP and found a positive additive interaction for 130 mmHg (RERI was 0.81 [95% CI 0.17; 1.45], p = 0.013), but not for 150 mmHg. Furthermore, we assessed interaction between SBP and the wGRS on a continuous scale, which resulted in a RERI of 3.72 (95% CI 0.55; 6.89), p = 0.02. There was no significant interaction (additive or multiplicative) between DBP and the wGRS in relation to UC outcomes.

The 20-year risk of any UC for 60-year old men was 2.4% for those with normal SBP (< 140 mmHg) with low wGRS, and 3.7% for those with elevated SBP (≥ 140 mmHg) with high wGRS. The corresponding 20-year risk for aggressive UC among 60-year old men was 0.78% and 1.33%, respectively.

## Discussion

In this prospective study, we confirmed a positive association between SBP and aggressive UC risk, and between a wGRS and UC overall and non-aggressive disease. Additionally, we found a positive additive interaction between SBP and wGRS in relation to aggressive UC, suggesting that the joint risk increase by high SBP and wGRS is greater than the sum of their individually contributing risks. Our results indicate that prevention and early treatment of hypertension in men with high genetic risk for BC, might efficiently prevent a substantial portion of lethal UCs.

The association between SBP and aggressive UC among men is consistent with findings from previous studies based on muscle invasiveness (NMIBC and MIBC)^12–15^. Speculatively, the association between SBP and aggressive UC and not with non-aggressive disease suggests that SBP may play a role in cancer progression rather than in cancer initiation. However, the reasons to why the association is only seen in men in previous studies, and only for SBP and not DBP, and whether this association is causal, remain unclear. In a previous much larger study of men in Sweden, we found a positive association between SBP and MIBC among never-smokers, in which any residual confounding by smoking, the main potential confounder in the association, should be minimal. In that study, the associations in never-smokers were stronger than in the full population, suggesting that the findings for BP in the present study could be slightly underestimated. However, potential biological mechanisms linking SBP and BC, and in particular aggressive BC, have not yet been elucidated. Studies for experimental science have speculated that the renin-angiotensin system may play a role in carcinogenesis^28,29^.

Prospective cohort studies have shown that GRSs can contribute to the risk of developing disease, thus, the consistent association between the wGRS and BC with previous studies was not surprising^30,31^. However, in previous studies, the wGRS was constructed from a smaller number of SNPs and the association was investigated with total BC, which combined both aggressive and non-aggressive tumors^30,31^. We further investigated the association separately for non-aggressive and aggressive tumors, where we found an association for non-aggressive UC, but not aggressive UC. These results are expected given that the BC GWAS was non-specific and was more likely to be based on non-aggressive or non-muscle invasive BC which make up about 75% of all UC, a more ideal investigation for the rarer aggressive or muscle-invasive BC would be to include BC GWAS based specifically on aggressive tumors as opposed to BC in general.

We found an additive interaction between SBP and wGRS in relation to aggressive UC both when using specific categories and on a continuous scale. For the specific categories, we used 130 mmHg, 140 mmHg and 150 mmHg as the cut-off points for SBP. When applying a cut point for SBP at 130 mmHg, 140 mmHg, the additive interaction with wGRS persisted, whilst it did not when applying a cut point at 150 mmHg. This interaction suggests that genetics and SBP may have a stronger joint effect than the sum of each risk factor individually in relation to aggressive UC. Furthermore, the excess risk that is due to the interaction between genetics and SBP, suggests that they may share common pathways that lead to aggressive UC^32^. The magnitude of RERI based on risk ratios is uninformative without knowledge of the baseline (background) risk of aggressive UC. However, only the direction, as opposed to the magnitude of RERI (based on risk ratios) is necessary to make conclusions regarding the public health relevance of the interaction^33,34^. Interaction on the additive scale is rarely studied in epidemiological studies, yet it is widely regarded to be a reflection on an underlying biological interaction^32,35^. While the biological insight provided by additive interaction may still be in question, its importance in public health is consensus, since it helps to identify the sub-group which is at most risk or will benefit most from an intervention^35^. This result should however, be interpreted with caution given that there were relatively few cases of aggressive UC and that the wGRS was likely based on GWAS less specific to this sub-group, increasing the likelihood of a false positive finding. Studies on gene-environmental interaction in relation to UC are common^4,8,18,36^, however, to our knowledge, there are no prior studies on the interaction between BP and genetics. The 20-year absolute risk for aggressive UC among 60-year old men with elevated SBP and high wGRS is nearly twice as high as those with normal SBP and low wGRS. While the relative risk of occurrences in these two groups is small in absolute terms, elevated blood pressure (hypertension) is a significant global health burden, especially in high income countries, and among men with increased genetic susceptibility to UC, prevention or early treatment of hypertension may potentially reduce the risk of lethal UC by half.

The main strengths of the study were the long and complete follow-up of the cohort, and use of histopathologically verified tumor data. Furthermore, the wGRS incorporated most SNPs discovered in GWAS of European ancestry to date. A main limitation of the study is the statistical power, which was reflected in the fewer number of cases in some of the sub-groups, especially among the aggressive UC sub-group, which may require a larger sample size to produce more informative results. Furthermore, although we had data on antihypertensive medication, which we consider to be an effect modifier or potentially a mediator in the relationship between BP and UC, we were unable to investigate associations separately by antihypertensive intake due to limited numbers in the antihypertensive user group.

In conclusion, our findings support an association between SBP and aggressive UC, between wGRS and UC overall and non-aggressive UC, and an additive interaction between SBP and wGRS in relation to aggressive UC. Elevated blood pressure is very common in high-income countries, and our findings suggest that prevention and early treatment of hypertension, particularly amongst men with high genetic risk for BC, could prevent a significant portion of lethal BCs and UCs. However, whilst our findings on interaction may provide such biological and public health insight, replication in larger studies is needed.

## Data Availability

Due to ethical and legal restrictions related to the Swedish Biobanks in Medical Care Act (2002:297) and the Personal Data Act (1998:204), data that support our findings are available upon request from the data access group of Malmo Diet and Cancer study by contacting Anders Dahlin (anders.dahlin@med.lu.se).
